# Organ-Chips Enhance the Maturation of Human iPSC-Derived Dopamine Neurons

**DOI:** 10.3390/ijms241814227

**Published:** 2023-09-18

**Authors:** Maria G. Otero, Shaughn Bell, Alexander H. Laperle, George Lawless, Zachary Myers, Marian A. Castro, Jaquelyn M. Villalba, Clive N. Svendsen

**Affiliations:** Board of Governors Regenerative Medicine Institute, Cedars-Sinai Medical Center, Los Angeles, CA 90048, USA; mgabriela.otero@cshs.org (M.G.O.);

**Keywords:** organ-chip, dopamine neurons, human, iPSCs, maturation

## Abstract

While cells in the human body function in an environment where the blood supply constantly delivers nutrients and removes waste, cells in conventional tissue culture well platforms are grown with a static pool of media above them and often lack maturity, limiting their utility to study cell biology in health and disease. In contrast, organ-chip microfluidic systems allow the growth of cells under constant flow, more akin to the in vivo situation. Here, we differentiated human induced pluripotent stem cells into dopamine neurons and assessed cellular properties in conventional multi-well cultures and organ-chips. We show that organ-chip cultures, compared to multi-well cultures, provide an overall greater proportion and homogeneity of dopaminergic neurons as well as increased levels of maturation markers. These organ-chips are an ideal platform to study mature dopamine neurons to better understand their biology in health and ultimately in neurological disorders.

## 1. Introduction

Human induced pluripotent stem cells (iPSCs) provide a powerful platform to better understand human biology and, further, patient-derived iPSCs can be used for faithful disease modeling [1,2,3,4]. However, traditional two-dimensional (2D) monocultures lack the 3D multicellular complexities that influence cells in the body [5,6,7]. In order for iPSCs to generate mature cellular phenotypes that resemble biology in health and in disease, it may be required to use 3D systems such as organoids and microfluidic organ-chip platforms, as recently reviewed in Sharma et al., 2020 [7,8,9,10].

The use of biocompatible materials combined with microengineering technologies have enabled the development of microfluidic organ-chips [11]. Organ-chips are designed for the growth of multiple organ-specific cell types in a fully integrated system, often with the different cell types being cultured in two separate compartments that are connected via a membrane containing microchannels. Since the body is not a static system, but rather perfused by flowing blood vessels, organ-chips provide a further advantage over 2D cultures as they enable fluid flow across the cells.

Dopamine neurons are critically involved in voluntary movement and various behavioral processes such as mood, reward, and addiction. Furthermore, changes in dopamine underlie several neurological disorders, including attention deficit disorder, schizophrenia and Parkinson’s disease (PD) [12,13,14]. While human dopamine neurons have been extensively cultured in 2D platforms [3,15,16,17], these neurons may lack the maturity to faithfully reproduce healthy and diseased states. Here, we differentiate human iPSCs into dopaminergic neurons and compare a 2D conventional culture system and an organ-chip system. Based on immunocytochemistry and single-nucleus RNA sequencing (snRNA-seq), we show that the organ-chip platform provides an increased maturation and more homogeneous culture of dopaminergic neurons. The development of a human iPSC-derived dopamine neuron organ-chip can provide the field with mature dopamine neurons, which can be used to better understand their biology in health and ultimately in disease.

## 2. Results

### 2.1. Astrocytes Increase Dopamine Neuron Survival in 2D Well and Organ-Chip Cultures

**Figure 1:**
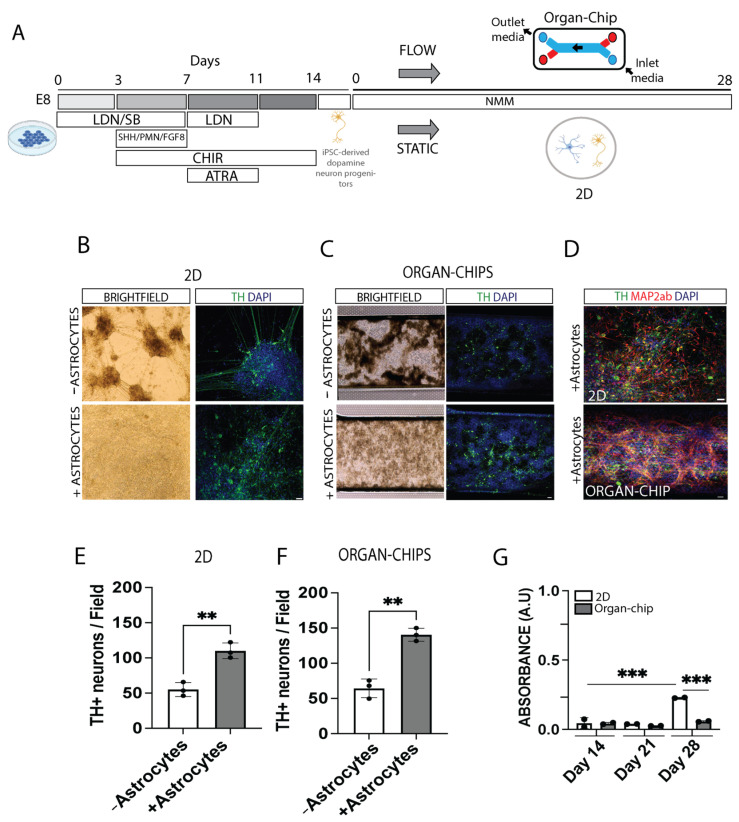
Human iPSCs can be differentiated in dopaminergic neurons. (**A**) Differentiation schematic for dopaminergic neuron cultures. (**B**,**C**) Representative images of brightfield and cultures stained with Tyrosine Hydroxylase (TH) (green) and DAPI (blue) after 28 days with and without human midbrain astrocytes in (**B**) 2D well cultures and (**C**) organ-chip. (**D**) Representative image of 2D well and organ-chip cultures stained with TH (green), MAP2ab (red) and DAPI (blue). (**E**,**F**) TH+ neurons quantification per field. n = 3 independent experiments. Student’s *t* test, ** *p* < 0.002. (**G**) Lactate dehydrogenase assay was performed on media from 2D well and organ-chip cultures at days 14, 21 and 28. n = 2 independent experiments with n = 6 organ-chips per experiment; n = 4 2D wells per experiment. Samples were quantified in duplicate and averaged. One-way ANOVA with Tukey post-test, *** *p* < 0.001. Error bars represent mean ± standard deviation (SD). Scale bar: 50 µm.

To establish how dopamine neurons may survive in 2D well or organ-chip cultures, human iPSCs were differentiated into dopamine neuron progenitors over 15 days and then collected and cryopreserved until subsequent thawing and seeding onto glass coverslips in conventional 2D wells or into the top channel of an organ-chip. Cells were maintained in neuronal maturation media, with media changes every 2 days for 2D wells and a constant flow of 20 µL/h for organ-chips. At day 28, samples were either fixed for immunocytochemistry or processed for analysis with snRNA-seq (Figure 1A).

**Figure 2:**
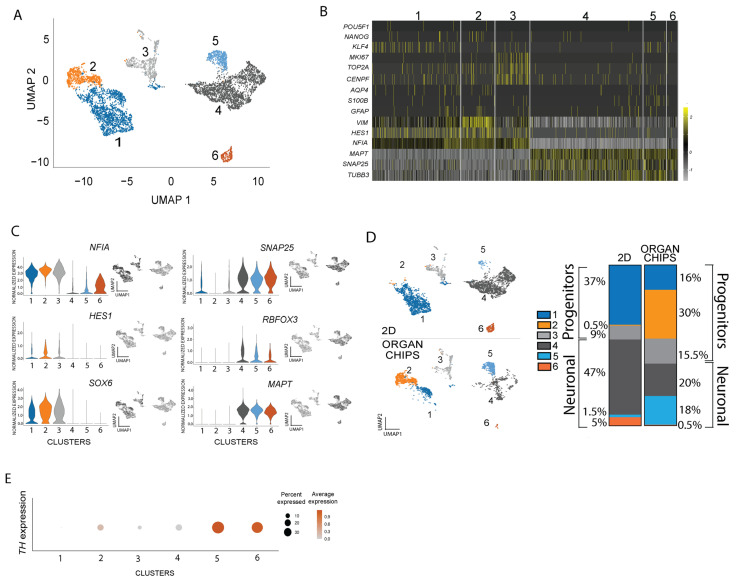
Different subtypes of dopamine neurons across clusters. (**A**) UMAP shows iPSC-derived dopamine 2D well cultures and organ-chips combined expression profile and clusters. (**B**) Heatmap shows the differential expression of pluripotency, proliferation, astrocyte, progenitor and neuronal markers. (**C**) Violin plots and UMAPs show differential expression of progenitor markers *SOX6*, *HES1* and *NFIA* and mature neuron markers *RBFOX3*, *MAPT* and *SNAP25*. (**D**) UMAP split cluster between 2D well and organ-chipcultures (left panel) and the proportion of each cluster in 2D well or organ-chip cultures (right panel). (**E**) Dot plot shows *TH* expression (color intensity) and percentage of cells expressing TH (dot size) in each cluster.

Phase images of live cells showed many clumps within both the 2D well cultures and organ-chips, and immunocytochemistry revealed the number of tyrosine hydroxylase (TH)-positive dopamine neurons was poorly distributed (Figure 1B,C, top panels). As astrocytes are known to play an important role in neuron maturation and stability [18], we co-cultured the dopamine neuron progenitors with commercially available (ScienCell Research Labs) primary human astrocytes isolated from the midbrain. Immunocytochemistry for TH alone, as well as co-staining for TH and the neuronal marker MAP2ab, showed that dopamine neurons co-cultured with astrocytes had a more even distribution throughout the 2D well cultures and organ-chips and increased fiber outgrowth (Figure 1B,C, bottom panels, Figure 1D). Quantification demonstrated a significant increase in the total number of TH-positive cells when co-cultured with astrocytes compared to without astrocytes, with a trend (*p* = 0.064) for more TH-positive cells in organ-chip cultures compared to 2D wells (Figure 1E,F—2D well culture with no astrocytes 55.18 ± 9.87 vs. with astrocytes 110.10 ± 11.13; organ-chip culture with no astrocytes 64.39 ± 13.29 vs. with astrocytes 140.5 ± 9.26). In order to quantify culture health over time, a lactate dehydrogenase (LDH) assay was performed, which showed that 2D well cultures at day 28 had significantly increased cell death compared to earlier time points and compared to organ-chip cultures (Figure 1G).

Surprisingly, even though astrocytes were seeded at the beginning of the experiment, very few glial fibrillary acidic protein (GFAP)-positive astrocytes were observed at day 28 (Supplementary Figure S1A). We hypothesized that the astrocytes had not survived due to the use of neuronal maturation media containing the mitotic inhibitor DAPT and lacking serum. To investigate this further, the primary astrocytes were maintained for 28 days in 2D well culture in either astrocyte-specific media or neuronal maturation media. GFAP-positive astrocytes survived in astrocyte media, yet this was greatly reduced in neuronal media, although there were some GFAP-negative, DAPI-stained cells (Supplementary Figure S1B). In order to assess astrocyte death overtime, astrocytes alone were maintained in neuronal media, and an LDH assay was performed. The results show a significant increase in astrocyte death by 28 days (Supplementary Figure S1C). As primary astrocytes appear to die overtime in neuronal media, it is likely that remaining GFAP-negative cells reflect other cells types, as the commercially available astrocytes were not sorted for purity. However, this cell subpopulation is expected only at low levels given the already dilute ratio of astrocytes plated in co-culture.

### 2.2. Dopamine Neuron Proportion and Homogeneity Are Increased in Organ-Chip Compared to 2D Well Culture

To further characterize and quantify dopamine neurons in 2D well cultures and organ-chips, we generated an additional set of dopamine neuron cultures with astrocytes (2D n = 2, organ-chips n = 2) (Supplementary Figure S2A,B) for snRNA-seq. We analyzed 5538 total nuclei (3548 nuclei in the 2D well cultures and 1990 nuclei in the organ-chips) from all cultures after quality filtering, with an average of 11,156 reads and 4227 genes detected per nucleus. Six distinct clusters were identified (Figure 2A). As expected, the differentiated cultures showed barely detectable expression of pluripotency markers *(POU5F1*, *NANOG* and *KLF4*) (Figure 2B). A few dividing cells based on the proliferation markers *MKI67*, *TOP2A* and *CENPF* were found in Cluster 2 (Figure 2B). Just a few cells with *AQP4*, *S100B* or *GFAP* expression were detected (Figure 2B), corroborating the lack of GFAP staining and further suggesting that the astrocytes may not survive in the organ-chips to the endpoint. Finally, in order to further analyze cell identity, clusters were categorized into progenitor and neuronal identities based on the expression of specific cell type markers (Figure 2C). The expression of dopaminergic progenitor markers *SOX6*, *HES1* and *NFIA* [19,20] identified three progenitor clusters, and the expression of neuronal maturation markers *RBFOX3*, *MAPT* and *SNAP25* [21] identified three neuronal clusters.

In order to separately evaluate the amount of neurons in 2D well cultures versus organ-chips (Figure 2D, left panel), the proportion of each cluster was quantified using the number of cells per cluster from the total amount of cells calculated (Figure 2D, right panel), showing that organ-chips had unique populations of progenitors and neurons. Despite some differences in the proportion of Clusters 1 and 3, the major difference between 2D well and organ-chip progenitors occurred in Cluster 2 (0.5% for 2D wells and 30% for organ-chips), which showed the greatest combination of floorplate markers *CORIN1*, *LMX1A* and *FOXA2* (Supplementary Figure S2C). This suggested the organ-chip environment was driving cells towards a dopaminergic fate. Single-cell RNA-seq analysis of mouse embryos has revealed two populations of dopaminergic progenitors that express the same markers (*LMX1A/B*, *OTX2*, *NR4A2)*, with one population generating dopaminergic neurons and the other glutamatergic neurons of the subthalamic nucleus (STN) [22]. We therefore evaluated the expression of STN markers, *BARLH1*, *DBX1*, *WNT8B* and *PITX2*, and we found barely detectable transcription levels compared with dopaminergic markers in either 2D well or organ-chip cultures (Supplementary Figure S2D). As these cultures showed expression markers for dopaminergic progenitors, we next quantified the TH expression (color intensity) and percentage of cells expressing TH (dot size) in each cluster (Figure 2E). This showed that ~30% of cells in Cluster 5 and ~20% of cells in Cluster 6 express TH. Collectively, the data suggest that even though 2D well cultures had the greater amount of total neurons, the proportion of *TH*-expressing neurons was highest in the organ-chips.

**Figure 3:**
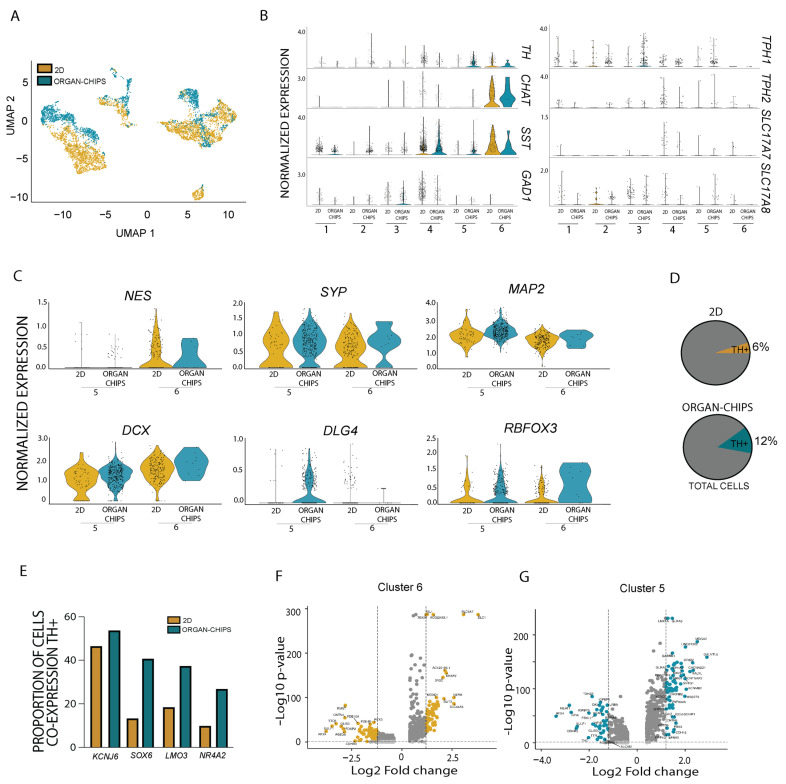
Dopamine neurons are greater in proportion and homogeneity in organ-chip vs. 2D well culture. (**A**) UMAP split between 2D well (gold) and organ-chip (green) cultures. (**B**) Violin plots show *TH*, *CHAT*, *SST*, *GAD1*, *TPH1*, *TPH2*, *SLC17A7* and *SLC17A8* expression across clusters. (**C**) Violin plots show *NES*, *DCX*, *SYP*, *DLG4*, *MAP2* and *RBFOX3* expression between Cluster 5 and Cluster 6 in 2D well (gold) and organ-chip (green) cultures. (**D**) Pie charts show the proportion of *TH*-positive neuron in 2D well (gold) and organ-chip (green) cultures along with no *TH* expression (grey). (**E**) Graph shows the % of co-expression of *TH* with *KCNJ6*, *SOX6*, *LMO3* and *NR4A2* in 2D well (gold) and organ-chip (green) cultures. (**F**,**G**) Volcano plots showing cluster markers in (**F**) Cluster 6 and (**G**) Cluster 5.

We next assessed the general expression profile of cells in the different cultures (Figure 3A). Since the conventional well platform generated a higher number of total neurons, yet a smaller proportion of *TH*-expressing neurons, we wanted to investigate the nature of these neurons. We therefore evaluated the gene expression level of neuronal markers such as choline acetyl transferase (*CHAT*, cholinergic neurons), somatostatin (*SST*, inhibitory neurons), glutamic acid decarboxylase (*GAD1*, inhibitory neurons), *TPH1* and *TPH2* (serotoninergic neurons) as well as *SLC17A7* and *SLC17A8* (glutamatergic neurons). While genes for inhibitory, serotoninergic and glutamatergic neurons were distributed throughout clusters in both 2D well and organ-chip cultures, *CHAT* and *SST* were highly enriched in the 2D well system in Cluster 6 (Figure 3B). Collectively, these findings suggest that while conventional well cultures have a higher overall level of neuronal cells, they are less specified and the majority appear to be cholinergic rather than dopaminergic.

A further assessment of the maturation markers between Clusters 5 and 6 showed that Cluster 6 (enriched in 2D wells) had a higher expression of the neural progenitor marker Nestin (*NES*) and the immature neuronal marker Doublecortin (*DCX*) compared to Cluster 5 (enriched in organ-chips) (Figure 3C). In contrast, Cluster 5 had increased gene expression for neuronal maturation markers like *DLG4*, *MAP2* and *RBFOX3*, while *SYP* remained stable between the cultures. Comparing the proportion of total *TH*-positive neurons showed that this was doubled in organ-chips (12%) compared to the 2D well system (6%) (Figure 3D). We next assessed the co-expression of TH with key dopaminergic markers. While a similar proportion of *TH*-positive neurons in 2D well and organ-chip cultures expressed the potassium inwardly rectifying channel subfamily J member 6 (*KCNJ6)* (2D well 46.5% vs. organ-chips 53.8%), far more TH-positive cells in organ-chip cultures co-expressed *SOX6* (2D well 13.4% vs. organ-chips 40.75%), *LMO3* (2D well 18.5% vs. organ-chips 37.4%) and *NR4A2* (2D 9.91% vs. organ-chips 26.9%), indicating that dopamine neurons in organ-chips showed elevated expression of several canonical A9 dopaminergic markers compared with conventional well cultures (Figure 3E). Finally, a volcano plot analysis was used to assess markers that were differentially expressed in Clusters 5 and 6 when compared to all cells. Significantly elevated genes in Cluster 6, which represented cells mostly from 2D well cultures, included non-*TH*-specific neuronal genes such as *ISL1* and *NEFM* (Figure 3F) which supports the findings for genes co-expressed in the TH neurons from 2D well cultures. Cluster markers for Cluster 5 (representing cells mostly from organ-chips) included developmental genes related to TH such as *LMX1A* [2] or the transcription factor *TMEFF2*, which has previously been identified as a midbrain neuron survival factor [23] (Figure 3G). These collective results demonstrate that organ-chips generate an increased proportion and a more homogeneous population of dopamine neurons.

## 3. Discussion

Human iPSCs can be differentiated into any tissue of the human body, but the accurate study of cell biology in health and disease requires a developmentally mature cell phenotype. While iPSC-derived 2D cultures have traditionally been used, this platform often lacks relevant multicellular complexity and faithful cell maturation [5,6,7]. In contrast, 3D cell culture models have the ability to better recapitulate the microenvironment and provide optimized cell maturation [7,8,9,10]. Given that dopamine neurons play many critical roles in the adult central nervous system, and as alterations in dopamine underlie several neurological disorders, the development of an in vitro culture system for mature dopamine neurons could be of great benefit to better understand their role in health and disease.

This study compared our published protocol [3] for dopamine neuron generation in a 2D well platform to an organ-chip system. We have now shown that the novel dopamine neuron organ-chip provides a more mature and homogenous population of dopamine neurons compared to conventional 2D well culture. Reduced neuronal survival was overcome by initial co-culture with human astrocytes, as utilized by others with long-term iPSC-derived dopamine neuron cultures [16]. Unlike conventional 2D well cultures that require routine media changes, the organ-chip system is equipped with an automated system that continuously feeds the cells with fresh media flow. We believe that the continuous media flow to replenish nutrients and remove metabolic waste may be a factor underlying the increased overall cell health as well as the generation of more mature neurons in the organ-chip platform, compared to the 2D well system with intermittent feeds to exchange consumed and fresh media. Critically, this automated system can ultimately permit the flow of compounds, such as drugs, through the chip to assess both their therapeutic function and ability to cross the blood–brain barrier (BBB). Indeed, we have already generated a human iPSC-derived BBB-chip model that can be used to screen drug transport and patient-specific drugs for personalized medicine or to recreate the vascular-neuron interface to model barrier disruption in disease [24,25,26].

While organ-chips, compared to 2D well cultures, appeared to have fewer neurons (39% in organ-chips vs. 54% in 2D) and a greater amount of progenitors (61% in organ-chips vs. 46% in 2D), the progenitors in the organ-chips showed higher co-expression of key floorplate markers that are known to give rise to mature dopaminergic neurons [15,21,27]. This suggests that organ-chips are poised for more specified differentiation into dopamine neurons and have an increased pool of *TH*-positive neurons. Though the neuronal Cluster 4 was highly enriched in 2D cultures, there was little expression of dopaminergic markers. The majority of *TH*-positive neurons were found in neuronal Clusters 5 and 6. However, Cluster 6 (mostly comprising cells from well cultures) was also enriched in additional neuronal markers. In contrast, Cluster 5 (mostly in organ-chips at 18% vs. 1.5% in 2D wells) contained more specific *TH* markers. In addition, maturation markers in those specific clusters showed some differences. The 2D well-enriched Cluster 6 showed higher gene expression for markers of neural progenitors and immature neurons. In contrast, the organ-chip-enriched Cluster 5 showed increased expression of the several post-mitotic neuronal maturation markers, and these were almost absent in 2D well cultures. Importantly, over 50% of the *TH*-expressing neurons in organ-chips co-expressed key A9 dopaminergic markers *KCNJ6* and *SOX6* [28,29]. In addition, organ-chip cultures had double the expression levels of dopaminergic maturation markers *LMO3* and *NR4A2* compared with 2D well cultures [20,21]. Collectively, these findings suggest that the organ-chip environment yields more mature neurons with an A9 dopamine fate [21].

The finding here that the organ-chip platform optimizes the generation of mature human iPSC-derived dopamine neurons compared to conventional well cultures mirrors the results from a study that showed enhanced maturity of human iPSC-derived dopamine neurons in an in vitro 3D alginate bead paradigm compared to 2D cultures [30]. Further, enhanced cell maturity with organ-chip cultures has been demonstrated across cellular models, for instance, with human iPSC-derived pancreatic beta cells showing that a pancreas-chip provided more biologically relevant cells to model pancreatic diseases [31] as well as demonstrations that human iPSC-derived 3D heart cell cultures enhance cellular maturation [10].

Patient-derived iPSCs provide a powerful in vitro model of human disease. However, robust phenotypes can be difficult to demonstrate with iPSC models of later-age onset diseases such as PD and Huntington’s disease [6,32,33]. This may partly be due to the lack of maturity for iPSC-derived neurons, which is supported by the emergence of phenotypes when neurons were matured via various methods [16,34,35]. The dopamine neuron organ-chip provides not only more mature dopamine neurons, but also markers specific to A9 dopamine neurons, the subtype in the substantia nigra that controls motor function and is primarily degenerated in PD, unlike the A10 subtype within the ventral tegmental area whose dysfunction associates with neuropsychiatric disorders [36]. For instance, *SOX6*-expressing neurons, which have been shown to be susceptible to PD-associated degeneration [37], show three-fold enrichment in the dopamine neuron organ-chip over well culture. Given the presence of A9-like mature dopamine neurons, there is a clear potential for the dopamine neuron organ-chip to ultimately be used to model PD and screen for new therapeutics. We are currently generating dopamine neuron organ-chips from patient-derived iPSCs and assessing phenotypes that we demonstrated for young-onset PD patients in 2D well culture [3]. While dopamine neurons on organ-chips have been used before to model synucleinopathies [25], neurons were from a commercially available source and were kept on the organ-chips for only 8 days, and no single cell analysis was performed to compare 2D well and organ-chip conditions. We believe that the culture of human iPSC-derived dopamine neurons for 28 days under constant flow provides significant advances for an ideal platform to ultimately model PD and uncover related phenotypes.

The organ-chip contains two separate channels, such that dopamine neurons and astrocytes can be co-cultured in one channel, and other cell types can be cultured in the second channel, for instance, adrenergic neurons involved in some Parkinsonian symptoms [38]. One area of great interest in PD is the role of the brain–gut axis. The current generation of a dopamine neuron organ-chip and prior generation of an intestinal-chip indicate that this platform is ideally poised to study the brain–gut axis role in PD with each relevant cell type in respective channels [39,40]. Finally, brain microvascular endothelial cells (BMECs) can be cultured in one channel in order to create a human BBB model to study drug transport [24]. While we have not yet looked at BMECs with dopamine neurons, we have previously shown that human iPSC-derived spinal cord neurons show increased neuronal differentiation and spontaneous neuronal activity when cultured in a spinal cord-chip with BMECs [41], for better modeling of diseases like amyotrophic lateral sclerosis.

Collectively, the organ-chip platform generates a larger proportion and more homogenous population of human dopamine neurons from iPSCs, provides media flow, and has the ability to co-culture multiple cell types in separate channels. With these attributes, the dopamine neuron organ-chip is ideal to study dopamine neuron biology and could ultimately be used to model dopamine-related neurological disorders, for instance, to gain a better understanding of PD and develop new therapeutics.

## 4. Material and Methods

### 4.1. iPSC Lines and Maintenance

Throughout all experiments, we used the iPSC line termed EDi044-A, which was derived from peripheral blood mononuclear cells (PBMCs) from a female from the Lothian cohort [42] at age 83 years with no signs of neuronal degeneration or cognitive decline. The iPSC line was generated by the Cedars-Sinai iPSC Core by nucleofecting parent cells with nonintegrating oriP/EBNA1 plasmids for reprogramming factors.

iPSCs were maintained in E8 medium on Matrigel and passaged every 5 days at split ratios from 1:6 to 1:12 using Versene (Gibco, Thermo Fisher Scientific, Waltham, MA, USA). Only iPSCs between passage 17 and passage 35 were used in this study. For differentiation, iPSCs were grown to ~80% confluency. Cells were singularized with Accutase (Millipore, Burlington, MA, USA/Sigma, St. Louis, MA, USA, SCR005) for 5 min at 37 °C and plated onto Matrigel-coated six-well plates (BD Biosciences, Franklin Lakes, NJ, USA) at 200,000 cells per cm^2^ (for a fully confluent monolayer) in E8 medium with Y27632 (StemGent/Reprocell, Beltsville, MD, USA).

### 4.2. Dopamine Neuron Differentiation

Human iPSCs were differentiated using our published protocol [3]. Briefly, 24 h after passaging, adherent iPSC colonies were induced to differentiate to Neuroectoderm in Stage 1 medium (50% DMEM/F12 and 50% neurobasal, N2, B27-vitamin A, LDN-193189 (LDN) and SB431542 (SB)). Stage 1 medium was changed each day (3 mL per well) for 3 days. For differentiation towards the rostral–caudal and the dorsal–ventral axes, cells were switched to Stage 2 medium (50% DMEM/F12 and 50% neurobasal, N2, B27-vitamin A, LDN, SB, purmorphamine (PMN), CHIR99021 (CHIR), Sonic hedgehog (SHH) and fibroblast growth factor 8 (FGF8)). Stage 2 medium was changed each day (3 mL per well) for 4 days. Medium was then switched to Stage 3 medium (50% DMEM/F12 and 50% neurobasal, N2, B27-vitamin A, LDN, CHIR and all-trans retinoic acid (ATRA)), which was changed each day (3 mL per well) for 4 days. Finally, cells were switched to Stage 4 neuronal maturation medium (50% DMEM/F12 and 50% neurobasal, N2, B27-vitamin A, brain-derived neurotrophic factor (BDNF), glial cell line-derived neurotrophic factor (GDNF), dibutyryl cyclic AMP sodium salt (dbCAMP), l-ascorbic acid (AA), γ-secretase inhibitor (DAPT), CHIR and transforming growth factor-β3), which was changed each day (3 mL per well) for 3 days. On day 15, dopamine neuron progenitors were dissociated to single cells using Accutase (30 min at 37 °C) and gently lifted. Dissociated cells were cryopreserved until further use. Dopaminergic maturation media (50% DMEM/F12 and 50% neurobasal, N2, B27-vitamin A, BDNF, GDNF, dbCAMP, l-ascorbic acid (AA), DAPT and transforming growth factor-β3) was used throughout the 28 days of experiment in either 2D well or organ-chip cultures.

### 4.3. Organ-Chip and 2D Well Cultures

The design of the organ-chip used for this model is based on previous protocols [24,41]. The chip is composed of a flexible polydimethylsiloxane (PDMS) elastomer that contains two closely opposed and parallel micro-channels separated by a porous flexible PDMS membrane (50 mm thick, with 7 μm diameter pores with 40 mm spacing, resulting in 2% porosity over a surface area of 0.171 cm^2^ separating the two channels). Organ-chips were coated with ER1 (Emulate, Boston, MA, USA) 1 mg/mL in ER2 (Emulate) and subsequently irradiated in the UVtron at 40% intensity for 120 sec. Organ-chips were then coated with Matrigel 1 mg/mL and incubated overnight at 37 °C and 5% CO_2_. Glass coverslips were coated with poly-l-ornithine and then laminin 40 μg/mL for at least 1 h at 37 °C. On the day of the experiment, cells were thawed and centrifuged 3 min at 1000 RPM, resuspended in neuronal maturation media with Rock inhibitor (10 μM) at 32,000 cells/μL and seeded into the top channel of the organ-chip or onto glass coverslips in 40 μL or 160 μL. After overnight incubation, organ-chips and coverslips were flushed with fresh media. For organ-chips, the media was flushed out through the outlet pore and connected to constant flow (Zoë^®^ culture module) of 20 µL/h neuronal maturation media until the end of the experiment. For glass coverslips, media was changed every 2 days. At 28 days post-plating on organ-chips or glass coverslips, media was removed, and cells were fixed in 4% paraformaldehyde at room temperature for 10–15 min.

### 4.4. Midbrain Human Astrocytes

Primary midbrain human astrocytes, which are not sorted for a purified astrocyte primary culture, were expanded in a T75 flask for two passages, according to manufacturer’s instructions (ScienCell Research Labs, Carlsbad, CA, USA, Catalog #1850). Cells were gently detached with 0.05% trypsin (BD Biosciences) for 2–4 min at room temperature and then resuspended at 100,000 cells/μL and seeded onto the coverslips or organ-chips at a 1:10 ratio of astrocytes/neurons.

### 4.5. Immunocytochemistry

Fixed cells on organ-chips or glass coverslips were washed in phosphate-buffered saline (PBS) and permeabilized in PBS Triton 0.2% for 10 min and blocked in PBS containing 5% donkey serum (Sigma) and Triton X-100 0.2% at 4 °C overnight. Primary antibodies were TH 1:300 (Novus Biologicals, Centennial, CO, USA, NB300-109), GFAP 1:1000 (Dako, Glostrup, Denmark, Z0334) and Map2ab 1:1000 (Sigma, M1406). Samples were washed three times in PBS and stained with species-specific Alexa Fluor 488- or Alexa Fluor 594-conjugated secondary antibodies (1:500 dilution, Invitrogen, Waltham, MA, USA) for 2 h at room temperature, followed by 4′,6-diamidino-2-phenylindole (DAPI) nuclear counterstain. Confocal z-stack images were acquired using an A1 microscope (Nikon, Tokyo, Japan) with 10× or 20× objectives and rendered using maximum-intensity projection through FIJI.

### 4.6. LDH Assay

Lactate dehydrogenase (LDH) was measured based on absorbance (A.U.) levels using an LDH cytotoxicity assay (Pierce, Rockford, IL, USA, kit #88953) following manufacturer’s instruction, along with the company-provided positive control. Briefly, media (50 μL) was removed from the organ-chip channel, 2D well cultures and astrocyte-alone cultures at days 14, 21 and 28. Within 1 h of collection, samples were quantified in duplicate and averaged, from n = 2 independent experiments with n = 6 organ-chips per experiment; n = 4 culture wells per experiment; and n = 1 well of astrocytes per experiment.

### 4.7. TH Neuron Quantification

The number of TH-positive neurons in 2D well and organ-chips cultures with and without astrocytes were counted in 3 independent experiments. For 2D well cultures, 7–10 fields per experiment were imaged and manually counted using FIJI. The number of TH was normalized to the chip area 1272 × 910 µm. For organ-chip cultures, 2 independent organ-chips per experiment were manually counted and normalized to the chip area 1272 × 910 µm.

### 4.8. Single Nuclei RNA-Seq Analysis

Nuclei isolation: The nuclei isolation protocol was modified from Corces et al. [43]. All steps were performed on ice, and all buffers were prepared less than 12 h before starting the nuclei isolation. Wide-bore Rainin LTS pipette tips were used exclusively throughout except where indicated. Frozen cell pellets were thawed on ice, resuspended in cold homogenization buffer (5 mM MgCl_2_, 3 mM Mg(Ac)_2_, 10 mM Tris pH7.8, 0.017 mM PMSF, 0.17 mM β-mercaptoethanol (β-ME), 320 mM Sucrose, 0.1 mM EDTA, 0.1% NP40, RNAse inhibitors and Protease inhibitors), and transferred to a 2 mL glass Dounce. Tissues were manually lysed with 50 strokes each of the “A” and “B” pestles. Homogenate was filtered using 70 μm Flowmi pipette tip filters and gently mixed 1:1 with a 50% Iodixanol solution (5 mM MgCl_2_, 3 mM Mg(Ac)_2_, 10 mM Tris pH7.8, 0.017 mM PMSF, 0.17 mM β-ME, RNAse inhibitors, Protease inhibitors and 50% Iodixanol). A centrifugation gradient was set up using 600 μL 40% Iodixanol solution (5 mM MgCl_2_, 3 mM Mg(Ac)_2_, 10 mM Tris pH7.8, 0.017 mM PMSF, 0.17 mM β-ME, RNAse inhibitors, Protease inhibitors, 160 mM Sucrose, 0.2 μg/μL OrangeG and 40% Iodixanol), 600 μL 29% Iodixanol solution (5 mM MgCl_2_, 3 mM Mg(Ac)_2_, 10 mM Tris pH7.8, 0.017 mM PMSF, 0.17 mM β-ME, RNAse inhibitors, Protease inhibitors, 160 mM Sucrose and 29% Iodixanol) and 800 μL of the 50% Iodixanol + Sample mixture (25% Iodixanol final concentration). This was centrifuged at 3000× *g* for 1 h with the breaks disengaged. Any debris on the top surface was removed, and 200 μL of the thin cloudy layer containing the nuclei was extracted with a regular-bore pipette tip, mixed with 1.8 mL PBS + 1% BSA solution with a wide-bore pipette tip, and centrifuged at 500× *g* for 10 min with breaks engaged. Then, 1.8 mL supernatant was removed, and the remaining 200 mL plus nuclei pellet was resuspended in 1.8 mL PBS + 1% BSA solution. The nuclei suspension was counted via hemocytometer and checked for nucleus quality. A portion of this suspension was then diluted to achieve the correct nuclei concentration for the 10× Chromium NextGEM 3′ protocol with a target of ~4000 nuclei per sample.

Single-nuclei library preparation and sequencing: The standard 10× protocol was used per the “Chromium NextGEM Single Cell 3′ Reagent Kits v3.1 User Guide, Rev D” (single index). Briefly, nuclei were resuspended in the master mix and loaded together with partitioning oil and gel beads into the chip to generate the gel bead-in-emulsion (GEM). The poly-A RNA from the nucleus lysate contained in every single GEM was retrotranscripted to cDNA, which contains an Ilumina R1 primer sequence, Unique Molecular Identifier (UMI) and the 10× Barcode. The pooled, barcoded cDNA was then cleaned up with Silane DynaBeads and amplified by PCR, and the appropriately sized fragments were selected with SPRIselect reagent for subsequent library construction. The amplified cDNA and sequencing libraries were quality checked on an Agilent 2100 BioAnalyzer (Agilent Technologies, Santa Cruz, CA, USA) using a High-Sensitivity DNA chip. Barcoded sequencing libraries were quantified via qPCR on a QuantStudio12k Flex (Thermo Fisher Scientific) system using the Collibri Library Quantification Kit (Thermo Fisher Scientific). The uniquely indexed libraries were pooled at equal ratio and sequenced on a NovaSeq 6000 (Illumina, San Diego, CA, USA) as per the Single Cell 3′ v3.1 Reagent Kits User Guide, with a sequencing depth of ~50,000 reads/cell. Raw sequencing data were demultiplexed and converted to FASTQ format using bcl2fastq v2.20.

Data analysis: Demultiplexed fastq files were run via CellRanger v6.1.2 using the “cellranger count” command with the “--include-introns” option using the precompiled CellRanger human reference sequence “v2020-A” provided on the 10× Genomics website. CellRanger output “filtered_feature_bc_matrix files” (barcodes.tsv.gz, features.tsv.gz and matrix.mtx.gz) were loaded into R (v4.2.1) via Rstudio (v2022.07.1, Build 554) and combined into one matrix file per sample with genes as rows (with both gene symbol and Ensmbl ID) and cells (nuclei) as columns. Each matrix file was then processed by summing all Ensmbl IDs with the same gene symbol to the gene symbol level via standard R matrix processing functions. The gene-summed matrices were loaded into the Seurat R package (v4.1.1), keeping all genes that were expressed in more than one cell.

Two-dimensional well and organ-chip samples were then merged into a single Seurat object (7086 total nuclei: 3958 2D well and 3128 organ-chip) and filtered by removing all cells with counts, genes, mitochondrial gene percentage and ribosomal gene percentage outside of three standard deviations from the mean (6739 total nuclei: 3880 2D well and 2859 organ-chip). The filtered Seurat object was normalized via the “SCTransform()” function using mitochondrial gene percentage as a regression variable. Principal component analysis (PCA) was run using 50 principal components (PCs). The number of “meaningful PCs” used in subsequent analyses was determined by comparing the actual contributed variance of each PC versus the hypothetical situation where each PC would contribute equally to the variance. Thirteen PCs contributed more variance than the calculated hypothetical equal-variance level and were considered “meaningful”. Next, the nuclei were clustered using the Seurat functions “RunUMAP()”, “FindNeighbors()” and “FindClusters()”. A resolution factor of 0.1 was used, resulting in the identification of six distinct clusters. As a final quality control step, clusters were inspected again for counts per nucleus, genes per nucleus, mitochondrial gene percentage, and ribosomal gene percentage. One cluster was removed due to low counts, low features, high mitochondrial gene percentage, and high ribosomal gene percentage, resulting in 5538 total high-quality nuclei remaining (3548 2D and 1990 organ-chip). These remaining high-quality nuclei were reprocessed as described above (SCTransform, PCA, RunUMAP, FindNeighbors, and FindClusters). The same number of PCs and resolutions were determined optimal (13PCs and a resolution of 0.1) and resulted in six clusters. For generation of UMAP plots, the RNA slot in the SCTransformed Seurat object was processed with the Seurat functions “NormalizeData()”, “FindVariableFeatures()” and “ScaleData()” using all genes present in each data set for the “features” option of “ScaleData()”. Cluster markers were determined using the “FindAllMarkers()” function. Differentially expressed genes in each cluster between the two groups (2D well vs. organ-chip) were determined using the “FindMarkers()” function. Volcano plots were generated using the “EnhancedVolcano” R package, and heatmaps were generated using the DoHeatmap() function.

### 4.9. Statistical Analysis

Significance testing for TH used Student’s *t* test and testing for LDH overtime used one-way ANOVA with Tukey post-test, with *p* < 0.05 values as significant and error bars representing standard deviation. Significance testing for snRNA-seq was calculated using the default Seurat method “Wilcox Rank Sum”, and only adjusted *p* values < 0.05 were considered significant.

## Data Availability

GEO accession number GSE239951.

## Supplementary Materials

The following supporting information can be downloaded at: https://www.mdpi.com/article/10.3390/ijms241814227/s1.
