# Supplementary material for: Organ-Chips Enhance the Maturation of Human iPSC-Derived Dopamine Neurons
Source: Int J Mol Sci. 2023 Sep 18;24(18):14227. doi: 10.3390/ijms241814227 (PMC10531789; doi:10.3390/ijms241814227)
Supplement: Supplementary file 1 [file ijms-24-14227-s001.zip › ijms-2562819-supplementary.pdf]

Supplementary Materials

Organ-Chips Enhance the Maturation of Human iPSC-Derived Dopamine Neurons

Maria G. Otero, Shaughn Bell, Alexander H. Laperle, George Lawless, Zachary Myers, Marian A. Castro, Jaquelyn M. Villalba and Clive N. Svendsen *

Board of Governors Regenerative Medicine Institute, Cedars-Sinai Medical Center, Los Angeles, CA 90048, USA; mgabriela.otero@cshs.org (M.G.O)
* Correspondence: clive.svendsen@cshs.org

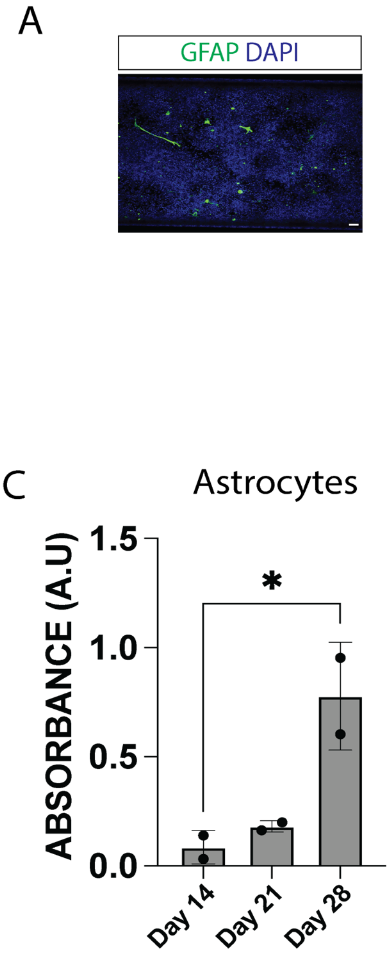

Figure S1. Astrocyte numbers are decreased with culture in neuronal media. (**A**) Organ-chip stained with GFAP and counterstained with DAPI at day 28. (**B**) Brightfield and immunocytochemistry of human midbrain astrocytes in neuronal maturation media or astrocyte media (bottom) after 28 days, with GFAP (green) and DAPI (blue). Scale bar 50

μm. (**C**) Lactate dehydrogenase assay was performed on media from 2D cultures of astrocytes alone in neuronal maturation media at days 14, 21 and 28. N=2 independent experiments with n=1 well of astrocytes per experiment. Samples were quantified in duplicate and averaged. Error bars represent mean ± standard deviation (SD). One-Way ANOVA with Tukey post-test *p<0.05.

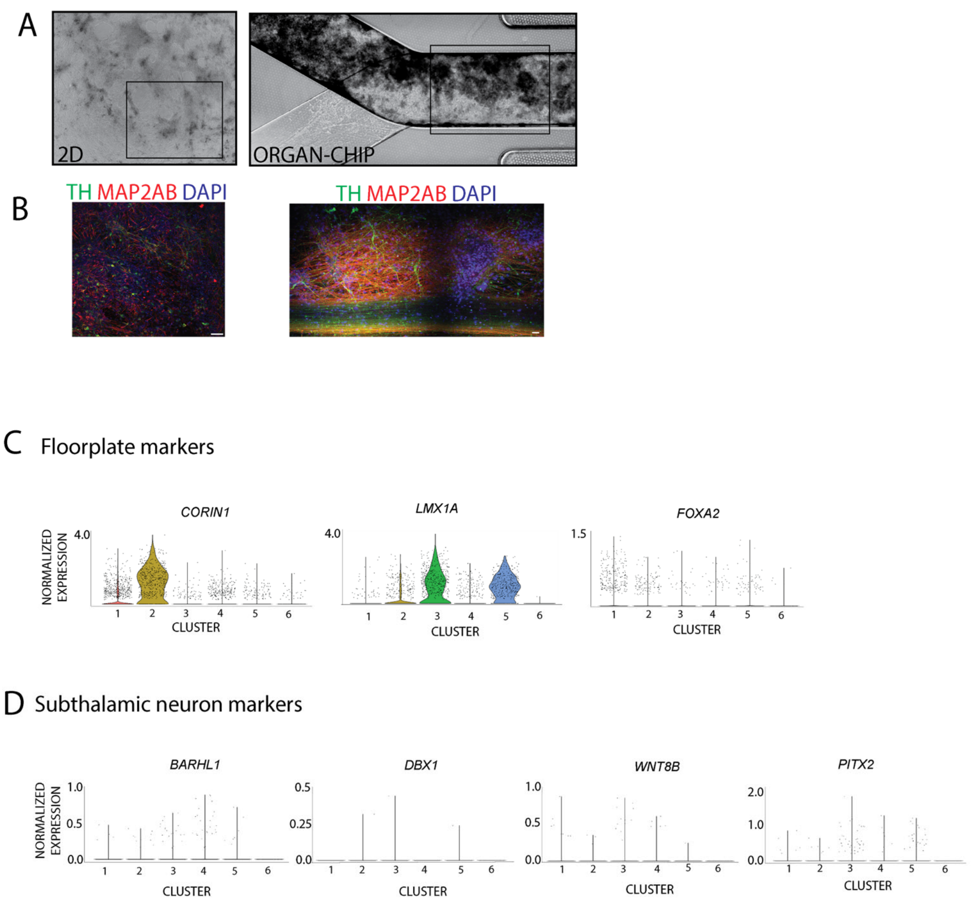

Figure S2. snRNA-seq analysis of 2D cultures and organ-chips. Additional 2D and organ chips used for the snRNA-seq experiment shown with (**A**) brightfield images of live cells and (**B**) immunocytochemistry for TH (green), MAP2ab (red) with DAPI counterstain (blue). Scale bar 50 μm. (**C**,**D**) Violin plots show the expression of (**C**) Floorplate markers (*LMX1A, FOXA2* and *CORIN1*) and (**D**) Subthalamic neuron markers (*BARHL1, DBX1, WNT8B* and *PITX2*).
